# Targeting Gene *C9orf72* Pathogenesis for Amyotrophic Lateral Sclerosis

**DOI:** 10.3390/ijms26094276

**Published:** 2025-04-30

**Authors:** Zhao Zhong Chong, Nizar Souayah

**Affiliations:** 1 Department of Neurology, New Jersey Medical School, Rutgers University, 185 S Orange, Newark, NJ 07103, USA; 2 Department of Neurology, New Jersey Medical School, Rutgers University, 90 Bergen Street DOC 8100, Newark, NJ 07101, USA

**Keywords:** amyotrophic lateral sclerosis, chromosome 9 open reading frame 72, hexanucleotide repeat expansion, RNA repeat, dipeptide repeat

## Abstract

Amyotrophic lateral sclerosis (ALS) is a fatal adult neurodegenerative disorder. Since no cure has been found, finding effective therapeutic targets for ALS remains a major challenge. Gene C9orf72 mutations with the formation of hexanucleotide repeat (GGGGCC) expansion (HRE) have been considered the most common genetic pathogenesis of ALS. The literature review indicates that the *C9orf72* HRE causes both the gain-of-function toxicity and loss of function of C9ORF72. The formation of RNA foci and dipeptide repeats (DPRs) resulting from HRE is responsible for toxic function gain. The RNA foci can interfere with RNA processing, while DPRs directly bind to and sequester associated proteins to disrupt processes of rRNA synthesis, mRNA translation, autophagy, and nucleocytoplasmic transport. The mutations of *C9orf72* and HRE result in the loss of functional C9ORF72. Under physiological conditions, C9ORF72 binds to Smith–Magenis chromosome region 8 and WD repeat-containing protein and forms a protein complex. Loss of C9ORF72 leads to autophagic impairment, increased oxidative stress, nucleocytoplasmic transport impairment, and inflammatory response. The attempted treatments for ALS have been tried by targeting *C9orf72* HRE; however, the outcomes are far from satisfactory yet. More studies should be performed on pharmacological and molecular modulators against *C9orf72* HRE to evaluate their efficacy by targeting HRE.

## 1. Introduction

Amyotrophic lateral sclerosis (ALS) is a fatal motor neuron neurodegenerative disease in adults, with the age of onset typically between 55 and 60 years of age [1]. Clinically, ALS is characterized by a focal onset of muscle weakness and a progressive weakness that spreads to all of the limbs and bulbar muscles, ultimately resulting in total paralysis and respiratory failure. No cure for ALS has been established, so pursuing effective therapeutic targets is currently the goal in the research community for ALS. Many risk factors, including age, sex, diet, smoking, and environment, may contribute to the progression of the disease. However, the underlying mechanisms that initiate the development and progression of ALS remain to be elucidated. In addition, genetics is also a critical factor that is involved in the pathological process of ALS. To date, over 100 gene mutations have been found to increase susceptibility to ALS. However, gene variants have been found in both familial and sporadic cases [2].

The common mutated genes that are associated with ALS are superoxide dismutase 1 (*SOD1*), TAR DNA binding protein 43 (TDP-43, *TARDBP*), fused in sarcoma (*FUS*), and chromosome 9 open reading frame 72 (*C9orf72*). The common feature of mutated proteins is that they can misfold and aggregate with other cellular components to form toxic aggregates that damage motor neurons. *SOD1* is the first gene found to be associated with ALS, accounting for about 20% of familial ALS cases. Mutations in *TARDBP* cause a dominant form of ALS and are found in about 1–4% of familial cases. However, the proteinopathy of its encoding protein, TDP-43, is also involved in the pathogenesis of sporadic ALS and may be linked to other gene mutations. Mutations in *FUS* were found in about 5% of familial ALS cases and about 1% of sporadic ALS cases. The gene variants of *C9orf72* and hexanucleotide repeat (GGGGCC) expansion (HRE) have been considered the most common genetic defects of familial ALS, accounting for about 20–25% of familial cases [3,4,5] and frontotemporal dementia [6,7]. In this regard, the C9orf72 gene and HRE have been extensively investigated for their roles in the pathogenesis of ALS and the therapeutic potential to target HRE.

The C9orf72 gene is on chromosome 9. The gene includes 11 exons. Exon 1, including 1a and 1b, is the non-coding exon, while exons 2 to 11 are coding exons. The hexanucleotide repeat falls in intron 1 between exons 1a and 1b. There are three variants. Variant 1, which includes non-coding exons 1a and 2–5, is the shortest; variant 2 includes exons 1b and 2–11, while variant 3 includes coding exons 2–11 and non-coding exon 1a. Only two isoforms are obtained; variant 1 encodes a 222-amino acid (aa), and variant 2 and variant 3a encode a 481-aa isoform [8]. The expression of human C9ORF72 has been found in the brain, spinal cord, and many other body systems [9]. Cellularly, C9ORF72 is highly expressed in CD14-positive monocytes and is up to sevenfold higher compared to the brain and other tissue cells [9].

Under normal physiological conditions, the number of hexanucleotide repeats is fewer than 11 copies [10], while the cut-off number of repeats for neurodegeneration has been generally considered to be more than 30 [11]. A meta-review analyzed the relationship between HRE and other neurodegenerative diseases and demonstrated that HRE over 30 was associated with various neurodegenerative diseases [12]. Increasing the *C9orf72* repeat size may promote DNA methylation in the *C9orf72* promoter, inhibiting transcriptional activity [13]. In ALS patients with *C9orf72* variants, HRE may reach hundreds or thousands of copies [14]. In some sporadic ALS patients of the Chinese population, 700–3500 repeats have been identified in 0.87% of the patients studied [15]. A much higher expansion rate was found in the European population [16]. However, the cut-off copy number was not always consistent among studies and remains to be determined [2].

The *C9orf72* HRE in ALS causes the formation of toxic repeats of RNA and dipeptide repeats (DPRs), which interact with other proteins to disrupt normal cellular processes, leading to a toxic gain of function [17,18,19]. The HRE formation also causes C9ORF72 to lose its normal physiological bioactivity, leading to loss of function. These mechanisms have been considered the major pathogenic contributors to the development of ALS.

## 2. Pathogenesis Due to Toxic RNA Foci

The sense (GGGGCC) and antisense (CCCCGG) of HRE repeats can undergo transcription to form RNA foci. The RNA foci are associated with RNA-binding proteins and splicing factors, disrupting RNA processing, such as splicing, transport, and translation. In ALS patients with *C9orf72* HRE, the antisense foci were highly expressed in Purkinje neurons of the cerebellum and motor neurons, which were included in aggregated inclusions [20]. The antisense repeat RNA (G2C4) sufficiently induces the pathological change of TDP-43, since antisense oligonucleotide (ASO) against the antisense repeat attenuates the dysfunction of TDP-43 in induced pluripotent stem cell (iPSC)-derived neurons [21]. The expression of antisense foci correlated with the accumulation of TDP-43 in the cytoplasm, a hallmark of ALS and one of its major pathogenic mechanisms [22].

**Figure 1:**
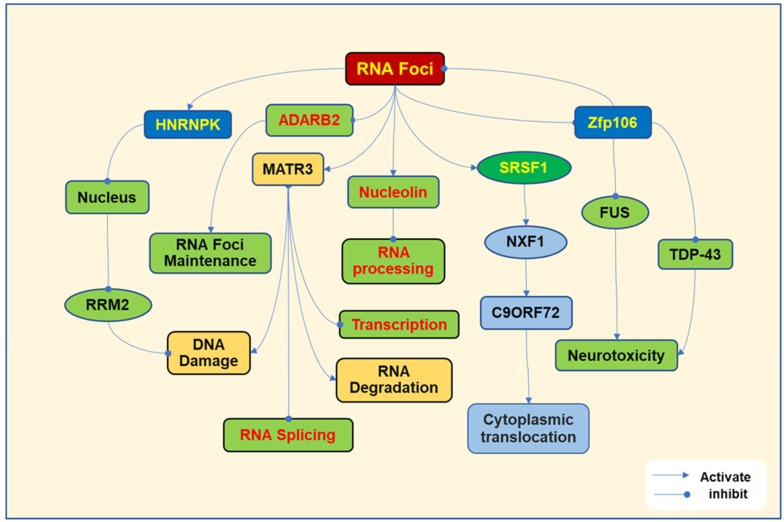
RNA repeats induce a gain of toxicity. RNA repeats undergo transcription, which results in the formation of RNA foci. RNA foci bind to heterogeneous nuclear ribonucleoprotein K (HNRNPK), which is translocated into the nucleus and inhibits ribonucleotide reductase regulatory subunit M2 (RRM2), leading to subsequent DNA damage. RNA foci target matrin3 (MATR3), regulating the response to DNA damage, impairing RNA transcription, disrupting RNA splicing, and resulting in RNA degradation. RNA foci also target adenosine deaminase RNA-specific B2 (ADARB2), which promotes the formation or maintains RNA foci. RNA foci are associated with serine/arginine-rich splicing factor 1 (SRSF1) and stimulate the nuclear RNA export factor 1 (NXF1), which promotes the nuclear export of C9ORF72. Zinc finger protein (Zfp)106 interacts with multiple RNA-binding proteins, including TDP-43 and FUS, to inhibit neurodegeneration. In addition, Zfp106 prevents the formation of RNA foci. RNA foci interact with nucleolin, impairing RNA processing, including rRNA processing, ribosome maturation, and mRNA translation.

RNN foci bind to different proteins, interfering with their activities and leading to cell toxicity. The commonly investigated RNA-binding proteins, including heterogeneous nuclear ribonucleoprotein K (HNRNPK), matrin3 (MATR3), adenosine deaminase RNA-specific B2 (ADARB2), serine/arginine-rich splicing factor 1 (SRSF1), zinc finger protein 106 (ZFP106), Ran GTPase-activating protein 1 (RanGAP1), and nucleolin, have been identified and play important roles in nucleoside transport, RNA splicing, and DNA/RNA binding (Figure 1).

RNA foci bind to HNRNPK to promote its translocation to the nucleus and inhibit ribonucleotide reductase regulatory subunit M2 (RRM2), damaging DNA [23]. The mutation in the RRM2 domain of TDP-43 in ALS promotes the progression of cellular aggregates in motor neurons [24]. MATR3 is a nuclear protein that regulates RNA metabolism, DNA regulation, and cell growth. RNA foci interact with MATR3, leading to the impairment of transcription, splicing, and the degradation of RNA [25]. MATR3 mutations have been found in sporadic ALS [26]. ADARB2 is an RNA-editing protein. RNA foci bind to ADARB2 to regulate RNA foci formation and maintenance [27]. RNA foci have been shown to cause the sequestration of ADARB2 [28]. SRSF1 is a nuclear protein that regulates RNA splicing, maintains RNA stability, and mediates nuclear export [29]. RNA foci bind to SRSF1 to stimulate the nuclear RNA export factor 1 (NXF1) to facilitate the C9ORF72 export to the cytoplasm [30]. SRSF1 depletion or inhibiting its interaction with NXF1 prevents the export of C9ORF72 transcripts from the nucleus and reduces the production of DRPs [31]. Zfp106 can interact with TDP-43 and fused in sarcoma (FUS) to prevent neurotoxicity [32]. FUS is a nuclear protein, the gene mutation of which has been implicated in the pathogenesis of ALS [33]. Zfp106 has been demonstrated to improve motor neuron survival in mice and a Drosophila ALS model. In addition, Zfp106 can prevent the formation of RNA foci and DPRs caused by G4C2 repeats [34]. Binding sense RNA repeat to RanGAP1 promotes nuclear export [35]. RNA foci also interact with nucleolin, impairing rRNA processing, disturbing the maturation of ribosomes, and inhibiting translation [36].

In conclusion, RNA foci bind to various proteins to induce aberrant functions and regulate the formation and maintenance of RNA foci, contributing to cytoplasmic aggregation, DNA damage, and abnormal RNA processing, which are involved in the pathogenesis of ALS (Figure 1).

## 3. Pathogenesis Due to Toxic DPRs

**Figure 2:**
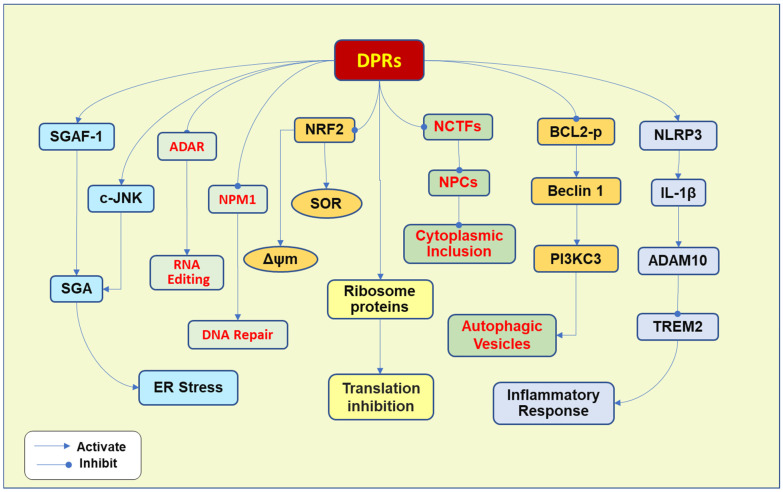
DPRs induce multiple pathogenic outcomes. DPRs interact with ribosomal proteins, resulting in translation inhibition. DPRs also interfere with RNA editing and DNA repair. p-PR can bind to the nucleolar protein nucleophosmin (NPM1) to inhibit the repair of DNA double-strand breaks and bind to adenosine deaminase acting on RNA (ADAR) to impair RNA editing. DPRs increase the endoplasmic reticulum (ER) stress by increasing the stress granule assembly (SGA) and aberrant deposition of stress granules in motor neurons by activating c-Jun N-terminal kinase (c-JNK) and increasing the transcription of stress granule assembly factor 1 (SGAF-1). Arginine-included DPRs increase the levels of superoxide radicals (SOR) and reduce mitochondrial membrane potential (Δψm) by inhibiting nuclear factor erythroid 2-related factor 2 (NRF2). Moreover, arginine-containing DPRs inhibit starvation-induced autophagy by inhibiting BCL2 phosphorylation (BCL2-p), leading to enhanced interaction between BCL2 and Beclin 1, which plays a crucial role in autophagy vesicle formation by activating phosphatidylinositol 3-kinase catalytic subunit type 3 (PI3KC3) and promoting the formation of the VPS34 complex. Furthermore, DPRs enhance the inflammatory response by promoting the release of inflammatory cytokines from microglia. P-GA activates the NLR Family Pyrin Domain Containing 3 (NLRP3) inflammasome to promote the production of interleukin-1β (IL-1β), while IL-1β activates A Disintegrin and Metalloproteinase 10 (ADAM10). ADAM1 mediates the cleavage of triggering receptors expressed in myeloid cell 2 (TREM2) in microglia. The downregulation of TREM2 leads to an increased inflammatory response. Toxic DPR proteins induce cytoplasmic aggregated inclusion formation by impairing the nucleocytoplasmic transport factors (NCTFs) and nuclear pore complex proteins (NPCs).

DPRs are formed by the non-ATG translation of the sense and antisense strands of the HRE [37]. The major DPRs include glycine–alanine (p-GA), glycine–proline (p-GP), glycine–arginine (p-GR), proline–alanine (p-PA), and proline–arginine (p-PR), which can directly bind and sequester other RNA processing proteins. p-GA is the most abundant DPR [38], while the arginine-included DPRs (p-GR and p-PR) are very toxic DPRs [39]. The overexpression of p-GA causes neurodegenerative phenotypes in the more transgenic mice, demonstrating progressive limb weakness [17]. p-GR induces a significant TDP-43 cytoplasmic accumulation and aggregation [40]. P-PR seems to play a lesser role in the pathogenesis of ALS, since it is much less abundant than p-GA in patients, although p-PR is most toxic in vitro [41]. The pathogenic mechanisms raised by DPRs involve multiple pathways (Figure 2).

### 3.1. Translational Inhibition and Nucleic Acid Repair

DPRs intervene in translational processes. Both p-GR and p-PR have been demonstrated to interact with ribosomal proteins to inhibit translation in both iPSC-derived human motor neurons and adult Drosophila neurons [42]. The p-GR proteins disturb the translation by impairing translation elongation and increasing cellular stress. In iPSC-differentiated neurons, p-GR has been shown to slow or stall the elongation, leading to ribosome collisions and ribotoxic stress response [43]. The arginine-containing dipeptides p-GR and p-PR inhibit upstream frameshift 1 (UPF1)-mediated nonsense-mediated decay in the brains of c9ALS/FTD patients and cultured cells [44].

DPRs have been demonstrated to interfere with RNA editing and DNA repair. p-PR targets nucleolar protein nucleophosmin (NPM1), which shuttles between the nucleolus and cytoplasm and mediates DNA repair in the nucleus. p-PR binds to and suppresses NPM1 to inhibit the repair of DNA double-strand breaks [45]. p-PR also binds to adenosine deaminase acting on RNA (ADAR). ADAR converts adenosine to an inosine in double-stranded RNA substrates [46]. There are three mammalian ADARs, including ADAR1, ADAR2, and ADAR3. p-PR is associated with AAR1p110 and ADAR2 to impair RNA editing [47]. ADAR2 specifically edits the GluR2 Q/R site, and ADAR2-mediated adenosine-to-inosine conversions at the GluR2 Q/R site are essential for the survival of mammalian organisms. However, the incomplete editing of this site was observed in the motor neurons of ALS patients [48], suggesting that ADAR editing impairment may play an essential role in the development of ALS.

### 3.2. Induction of Oxidative Stress

DPRs also induce elevated oxidative stress. P-GR increases the stress granule assembly and aberrant stress granule deposition in motor neurons [49] by activating c-Jun N-terminal kinase (c-JNK) and increasing the transcription of stress granule assembly factor 1 (SGAF-1) [50]. Moreover, p-GR binds to mitochondrial ribosomal proteins, impairing mitochondrial function and increasing the production of free radicals in iPSC-derived *C9orf72* motor neurons [51], while arginine-included DPRs promote the production of superoxide radicals and lower mitochondrial membrane potential by reducing the expression of nuclear factor erythroid 2-related factor 2 (NRF2) [52]. NRF2 binds to antioxidant response elements (AREs) to regulate the expression of antioxidant genes. Downregulation of NRF2 results in the reduction of antioxidants, leading to an increase in oxidative stress. p-PR has been shown to induce nucleolar stress and increase the transcription of p53, promoting neurotoxicity [53]. p-GA proteins promote the formation of cytoplasmic inclusions of filaments in primary neurons, stimulate the activation of caspase-3, impair neurite outgrowth, and increase stress granules of the endoplasmic reticulum (ER) [54]. ER stress is an important player in maintaining protein homeostasis, and elevated ER stress promotes the cytoplasmic accumulation and aggregation of abnormal protein inclusions that contribute to the pathogenesis of ALS [55]. DPRs, such as p-GA, p-GR, and p-GP, were reported to interrupt the transportation of secretory proteins from the ER to the Golgi apparatus in neuronal cells, leading to Golgi fragmentation and ER stress [56]. ER stress inhibition protects against p-GA-induced neuronal toxicity [38], suggesting that p-GA induces neurotoxicity, at least partially, through increasing ER stress.

### 3.3. Inhibition of Ubiquitin-Proteasome and Autophagy

p-PR has been demonstrated to inhibit ubiquitin–proteasome in rat spinal cord neuron cultures, resulting in an accumulation of ubiquitylated substrates and neuronal death [57]. In human and Drosophila melanogaster (*D. melanogaster*) brain tissues, p-PR was shown to bind to and impair the proteasome, accumulating lysosomes and ubiquitinated proteins [58].

Arginine-including DPRs, including both p-GR and p-PR, also strongly inhibit the autophagic process by inhibiting the phosphorylation of protein Bcl-2, promoting the interaction between Bcl-2 and Beclin 1 [59], the mammalian ortholog of the yeast autophagy-related gene 6, which increases autophagy vesicle formation by activating phosphatidylinositol 3-kinase catalytic subunit type 3 (PI3KC3), contributing to VPS34 complex formation. The inhibiting effects of p-GA on proteasomal processing were illustrated, since increasing the proteasome protein can attenuate the toxicity of p-GA [60].

### 3.4. Induction of Inflammation

DPRs also enhance the inflammatory response by promoting cytokine release from microglia and increasing apoptotic neurotoxicity. p-GA protein can activate the NLR Family Pyrin Domain Containing 3 (NLRP3) inflammasome in microglia to increase the production of interleukin-1β (IL-1β). IL-1β can activate A Disintegrin and Metalloproteinase 10 (ADAM10) to increase the cleavage of triggering receptors expressed in myeloid cells 2 (TREM2) [61], which plays an important role in the anti-inflammatory response in microglia [62]. TREM2 interacts with the tyrosine motif binding protein TYROBP to form a complex, activating immune cells to induce an inflammatory response. The ADAM10-induced downregulation of TREM2 leads to an increased inflammatory response. p-GR has also been demonstrated to activate the inflammasome in microglia and macrophages and increase the release of IL-1β [63].

### 3.5. Impairment of Nucleocytoplasmic Transport (NCT)

Moreover, DPRs affect the nuclear membrane and impair NCT. Toxic p-GR protein mediates cytoplasmic TDP-43 accumulation and induces aggregated inclusion formation, including TDP-43. The underlying mechanism may be due to the p-GR-induced dysfunction of NCT and the nuclear pore complex, leading to the TDP-43 translocation to the cytoplasm. p-PR directly binds to several nuclear transport components, including importin α (Impα), nuclear exportin receptor CAS, and GTPase-activating protein (RanGAP) [64]. Application of repeat-targeting ASOs lowers p-GR-induced TDP-43 pathology and neurodegeneration in mice [40]. p-GA also promotes cytoplasmic retention of TDP-43, which is ameliorated by anti-GA antibodies [65]. The effects of p-GA may be associated with its inhibition of proteasome activity, since overexpression of the proteasome protein 26S proteasome non-ATPase regulatory subunit 11 (PSMD11) or an increase in proteasome activity by rolipram application reduces p-GA-induced TDP-43 pathology [65].

### 3.6. The Dysfunction of Synapses

In motor neurons, DPRs interfere with synaptic function and reduce the expression of soluble human retinal gene-4 (HRG4), the human homolog of the Caenorhabditis elegans (*C. elegans*) gene uncoordinated-119 (Unc119), impairing the branching and maintenance of axons, leading to motor neuron degeneration [66]. Overexpression of p-GA reduces synaptic proteins, including presynaptic protein synaptophysin, synapsin 1 and 2, and synaptosome-associated protein 47, as well as the postsynaptic proteins postsynaptic density protein 95, gephyrin, and Homer1 in primary neuron cultures [67]. p-GA has been shown to sequester HRG4, impairing axonal protein trafficking and synaptic signal transduction [65]. In addition, p-GA disrupts the proteasomal degradation process, reduces the expression of synaptic vesicle-associated protein 2 (SV2), and impairs the influx of calcium and the release of synaptic vesicles, resulting in the dysfunction of synapses [68]. p-PA can increase membrane persistent sodium currents induced by the Nav1.2/β4 sodium channel complex, increasing synaptic hyperexcitability and leading to the death of motor neurons in *C9orf72* ALS [69].

As discussed above, DPRs induce multiple pathways implicated in ALS’s pathogenesis. DPRs interact with various proteins, leading to inhibition of translation, ER stress, failure of DNA repair, and autophagic impairment, which promote cytoplasmic accumulation and aggregation and motor neurodegeneration (Figure 2).

## 4. Pathogenesis Due to the Loss of C9ORF7 Function

**Figure 3:**
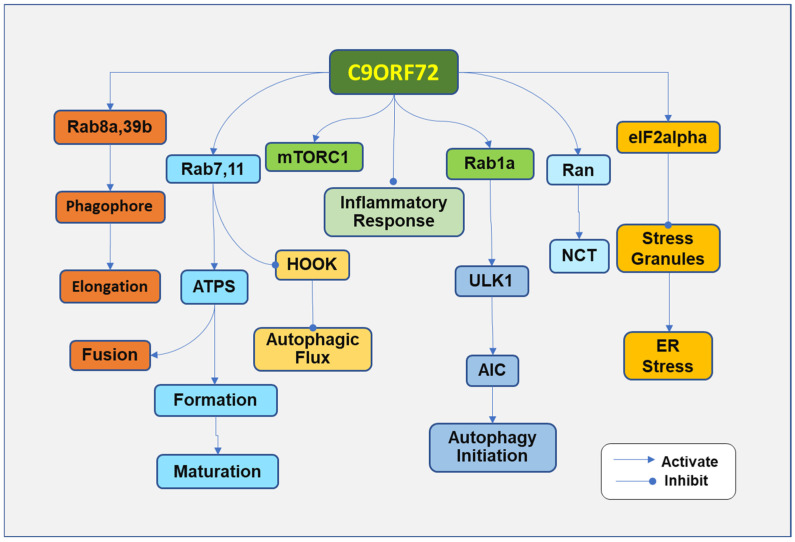
Some C9ORF72 functions, the impairment of which has been associated with amyotrophic lateral sclerosis. C9ORF72 mediates the autophagic process in many ways. C9ORF72 complex binds to Rab1a GTPase to control autophagy initiation by regulating the trafficking of the Unc-51-like kinase 1 (ULK1) to the autophagy initiation complex (AIC). The C9ORF72 complex interacts with Rab8a and Rab39b GTPase to regulate autophagic flux, which may be involved in phagophore elongation. C9ORF72 regulates vesicle trafficking of autophagy via Rab7 and Rab11 GTPases. Rab11 mediates autophagosome (ATPS) formation and maturation, promotes endosome and autophagosome fusion, and mediates autophagic flux by removing Hook from late endosomes. C9ORF72 maintains the gradient of Ran-GTPase (Ran) between the nucleus and cytoplasm, which guarantees the efficiency of nucleocytoplasmic transportation (NCT). In addition, C9ORF72 interacts with eIF2alpha to suppress the formation of stress granules, which lead to endoplasmic (ER) stress. However, the C9ORF72 complex may negatively regulate autophagy by activating the mammalian target of rapamycin complex 1 (mTORC1). C9ORF72 regulates Rag GTPase, which recruits the mTORC1 to the lysosome to sense nutrients under nutrient-rich conditions. Activation of mTORC1 inhibits autophagosome elongation, autophagosome maturation, and termination.

The functional loss of C9ORF72 can occur with *C9orf72* variants. Under physiological conditions, C9ORF72 forms a protein complex by binding to Smith–Magenis chromosome region 8 (SMCR8), which further associates with WD repeat-containing protein (WDR41) [70,71]. The C9ORF72-SMCR8-WDR41 complex regulates the autophagic process, membrane trafficking, and inflammation, which are implicated in ALS (Figure 3). The complex regulates lysosome homeostasis/autophagy and is associated with the activity of the mammalian target of rapamycin complex 1 (mTORC1) at lysosomes, in addition to many other bioactivities [72,73]. C9ORF72 has been demonstrated to regulate translation by interacting with eukaryotic initiation factor 2 subunit alpha (eIF2α). C9ORF72 deficiency resulted in a decreased interaction between eIF2α and eIF2B5, leading to the inhibition of translation [74]. The variants of *C9orf72* with the formation of HRE result in transcriptional repression, downregulation of C9ORF72, and impairment of C9ORF72 physiological functions [75]. Although C9ORF72 impairment is implicated in the pathogenic loss of function, its contribution to the development of ALS may be less than that of toxic gain of function. Reduced levels of C9ORF72 protein have been demonstrated to increase stress sensitivity and induce the degeneration of motor neurons in C. elegans and zebrafish [75,76]. However, the C9ORF72 loss was not found in mice with the phenotype of ALS or FTD [77].

### 4.1. Impairment of Autophagy

C9ORF72 deficiency impairs the autophagy–lysosome clearance pathway, alters microglial responses, and aggravates neuroinflammation [78,79]. Loss of C9ORF72 or SMCR8 causes the lysosome to swell and the impairment of mTORC1 responses to the changes in the availability of amino acid, leading to the failure of the autophagy–lysosome system and the deposition of TDP-43 aggregation [80].

The C9orf72 gene variants and their protein deficiency impair the autophagic process. Autophagy is a lysosome-dependent cleaning process that clears the intracellular aggregates. The C9ORF72 complex regulates multiple steps of the autophagic process. The impairment of autophagy has been observed in neurons with depletion of C9ORF72, promoting aggregates of TDP-43 with p62 proteins [81]. p62 is a scaffold protein and is involved in the ubiquitin-mediated process. Mutations in *sequestosome 1*, which encodes p62, have been found in ALS patients [82]. In C9ORF72-associated ALS, the colocalization of p62 and phosphorylated TDP-43 was found in cytoplasmic inclusions [83]. The complex of C9ORF72 and p62 has been associated with the use of arginine methylation to eliminate stress granules by autophagy [84].

C9ORF72 has been shown to regulate the initiation of autophagy. C9ORF72 interacts with Rab GTPases, which regulate the conversion of GTP-bound to GDP-bound proteins. The C9ORF72-SMRC8-WDR41 complex can affect Rab GTPase-activating proteins (GAPs), which catalyze GTP to form GDP [78], while the C9ORF72 protein catalyzes GDP back into GTP [85]. The switch between GTP-bound and GDP-bound Rab GTPases regulates several other proteins involved in the autophagy process. C9ORF72 binds to Rab1a GTPase [86] to control autophagy initiation by regulating the trafficking of the Unc-51-like kinase 1 (ULK1) autophagy initiation complex [87]. C9ORF72 deficiency impairs the formation of the initiation complex.

The C9ORF72 complex regulates autophagic flux by regulating Rab8a and Rab39b GTPase, which may be involved in phagophore elongation [88]. In *C9orf72* ALS motor neurons and *C9orf72* knockout iPSCs, lysosomes became swollen and less motile, and autophagic flux was slowed down [17],

C9ORF72 regulates vesicle trafficking of autophagy via Rab7 and Rab11 GTPases. Rab11 mediates autophagosome formation and maturation [89,90], promotes the endosome and autophagosome fusion, and mediates autophagic flux by removing the Hook from late endosomes. Since the Hook negatively regulates the maturation of endosomes, the removal of the Hook improves autophagic flux [91]. Rab7 is involved in the maturation of autophagic vacuoles and increases the colocalization of C9ORF72, Rab7, and Rab11 in motor neurons of C9ORF72 ALS [92].

C9ORF72 may also regulate endosome maturation, accelerating its fusion with lysosomes for degradation. In motor neurons differentiated from iPSCs derived from *C9orf72* ALS patients, impairment in endosome maturation was observed [17]. However, minimal effects on other autophagic steps suggest that C9ORF72 deficiency plays less of a role in regulating the impairment of autophagy compared with a gain of functional toxicity.

Interestingly, C9ORF72 has been associated with the mammalian target of rapamycin (mTOR) to regulate autophagy negatively. The mTOR functions through mTORC1. mTORC1 is composed of several proteins, including mTOR, the regulatory-associated protein of mTOR, the proline-rich Akt substrate 40 kDa, the lethal mammalian Sec13 protein 8, and the DEP domain-containing mTOR interacting protein [93,94]. The activated mTORC1 functions by activating p70 ribosome S6 kinase and eukaryotic initiation factor 4E-binding protein 1. C9ORF72 complex regulates Rag GTPase, which recruits mTORC1 to the lysosome to sense nutrients under nutrient-rich conditions [95]. Activation of mTORC1 inhibits autophagy in multiple steps, including autophagosome elongation, autophagosome maturation, and termination [96]. Active Rag GTPases can redistribute lysosomal mTORC1 and improve mTORC1 activity in cells with C9ORF72 deficiency [97]. In addition, C9ORF72 deficiency decreases the activation of mTORC1, increasing the expression of transcription factor EB (TFEB), which regulates lysosomal and autophagy genes in both C9ORF72-deficient animals and cells [97]. At this point, C9ORF72 seems to regulate autophagy negatively. The condition may be caused by the long-term deficiency of C9ORF72, which reduces mTOR activity and subsequently increases TFEB expression, improving cellular lysosomal capacity and autophagic flux [97]. The association between C9ORF72 and mTOR may partially explain the weak influence of C9ORF72 deficiency on the autophagic process compared with that of loss-of-function toxicity.

### 4.2. Dysfunction of NCT

Loss of C9ORF72 also disrupts NCT. Under physiological conditions, proteins or RNAs are transported between the nucleus and the cytoplasm by NCT. The gradient of Ran-GTPase between the nucleus and the cytoplasm guarantees the efficiency of NCT [98]. The HRE formation disrupts the Ran-GTPase gradient [99]. C9ORF72 deficiency impairs the Ran-GTPase gradient, disrupting NCT function by increasing importin β-1 granules and disrupting its association with the nuclear pore complex [99]. Cellular stress was reported to push the distribution of critical NCT factors into stress granules, leading to NCT impairment. Further observation indicated that the knockout of Ataxin-2 to inhibit stress granule assembly improved NCT function [81]. Moreover, C9ORF72 deficiency increased the expression of importin β-1 granules, induced its colocalization with stress granule assembly factor 1 (G3BP1) and K63-ubiquitin, and resulted in its interaction impairment with the nuclear pore complex [99].

### 4.3. ER Stress

The deficiency of C9ORF72 causes the dysfunction of ER stress. C9ORF72 has been demonstrated to interact with eIF2alpha to suppress the formation of stress granules [74]. In *C9orf72* HRE motor neurons, increased stress granule formation was observed [49]. In neurons differentiated from iPSCs derived from fibroblasts of *C9orf72* HRE patients, neuronal survival was significantly decreased, correlated with the imbalance in Ca^2+^ homeostasis. Moreover, in these neurons, the expression of the antiapoptotic protein Bcl-2 was decreased, accompanied by an increased ER stress [49].

### 4.4. Inflammation

Inflammation is implicated in ALS. In ALS patients, the variance of inflammatory cytokines, including granulocyte colony-stimulating factor, IL-2, IL-9, IL-4, IL-7, IL-17, IL-13, IL-6, IL-1β, and tumor necrosis factor in cerebrospinal fluid (CSF), was observed. More importantly, the changes were significantly associated with the peripheral neutrophil-to-lymphocyte ratio and disease progression rate score [100]. In ALS patients, inflammatory chemokine monocyte chemoattractant protein-1 (MCP-1) and IL-8 in CSF were also elevated [101].

C9ORF72 can regulate inflammatory response; C9ORF72 deficiency results in increased inflammation [102]. In *C9orf72* HRE-expressed microglia, LPS induced a consistent expression and release of matrix metalloproteinase-9 (MMP9), demonstrating toxic effects on co-cultured motor neurons. The application of an MMP9 inhibitor significantly attenuated neurotoxicity in motor neurons [103]. Loss of C9ORF72 causes lysosome defects, increasing the activation of the Janus kinase/signal transducers and activators of transcription (JAK/STAT) and increasing JAK/STAT-dependent inflammation.

Although loss of function may not be the significant pathogenesis of ALS, loss of C9ORF72 aggravates the impairment of the autophagic process, causes the dysfunction of nucleus–cytoplasm transportation, induces ER stress, and promotes inflammatory response, contributing to the development of ALS.

As discussed above, various mechanisms have been involved in the pathogenesis of ALS with C9ORF722 loss of function. However, animals often do not show ALS phenotypes after *C9orf72* knockout, since loss of function is not a major factor that contributes to the development of ALS. More complicated pathogenic factors are involved in the pathological development of ALS [8]. For example, the toxic gain of function plays a more important role than the loss of function. Moreover, C9ORF72 regulates immune homeostasis and an autoimmune response, and the loss of C9ORF72 is more likely to lead to increased systemic inflammation and autoimmune response, not neurodegeneration [104].

## 5. Targeting C9orf722 HRE for Potential Therapeutic Strategies

The pathogenesis of *C9orf72* HRE has been extensively investigated, demonstrating that multiple mechanisms under HRE were associated with the development of ALS. Either RNA repeats and DPRs or the loss of C9ORF72 significantly impacts the pathological processes of ALS, including RNA splicing, defective transport, and loss of stability. The deficiency of C9ORF72 causes increased stress in cells, impaired cytoplasmic aggregation, and decreased cellular ability to clear the aggregated inclusion.

The pathological processes involve many aberrant functional alterations of signal proteins, which could be considered potential targets for developing therapy for ALS. However, the complicated nature of the mechanisms involved should be noticed. For example, although the impairment of autophagy in ALS was illustrated by most investigations, either increased or decreased autophagy functions due to C9ORF72 deregulation have been identified. More investigations are needed to fully understand the pathogenesis of the C9ORF72 abnormality in the development of ALS. Nevertheless, the potential therapeutic effects of targeting *C9orf72* HRE have been extensively investigated for ALS.

### 5.1. Targeting RNA Repeats and DRPs

#### 5.1.1. ASOs

ASOs, small DNA sequences that reduce the expression of target genes, have gained attraction for antagonizing ALS-associated mutants or toxic transcripts. ASOs targeting *C9orf72* RNA have been demonstrated to prevent the neurodegeneration in *C9orf72* D. melanogaster and reduce the expression of sense RNA foci and DPRs in the *C9orf72* mouse model. In addition, RNA interference or small molecules have also shown some promise in reducing abnormal RNA foci and nucleotide repeats [105,106,107]. However, clinical trials have not demonstrated any prominent benefit. Recently, a phase I trial for BIIB078, an ASO against *C9orf72* sense RNA, failed to show any beneficial effects on clinical outcomes when compared to the placebo group [108]. In contrast, BIIB078 was shown to increase neurofilament levels in the CSF. BIIB078 was designed to target the sense HRE-containing C9ORF72 transcripts (encoded by exon 1a). The failure may indicate that targeting the exon 1a-encoded transcript is not sufficient. A stereopure ASO, WVE-004, which targets variants 2 and 3 transcripts, has been reported to dose-dependently and selectively reduce RNA repeat transcripts in motor neurons derived from patients with *C9orf72* HRE [109]. Although WVE-004 significantly reduced p-GP, no clinical benefits were observed after six months of treatment. ASOs with phosphodiester inter-nucleoside linkages can increase the patient’s tolerability, and repeated intrathecal mixed ASOs in a single patient with mutant *C9orf72* HRE significantly reduced the levels of p-GP in CSF [110].

The doses, ASO designs, and patient conditions may all affect the outcomes of clinical trials. The disease-developing stage may also be an essential factor that influences the effectiveness of ASOs. More advanced diseases with irreversible pathological status might be resistant to intervention. Nonetheless, the use of ASOs is still holding hope in the treatment of ALS, given the roles of *C9orf72* HRE in the development of ALS. Further insight into the mechanism and better design of more effective ASOs might be the future direction toward better outcomes.

#### 5.1.2. Gene Editing (CRISPR/Cas9)

The application of CRISPR/Cas9 construction in primary cortical neurons and brains of *C9orf72* mouse ALS models successfully mediated the excision of HRE, leading to reduced RNA foci and DPR inclusions [111]. In iPSCs derived from *C9orf72* HRE ALS patients, restoring the normal C9orf72 gene by CRISPR/Cas9 restored diminished pathological phenotypes [112]. The CRISPR/Cas13d system was also used to target G4C2 repeat RNA. The experiment significantly reduced the translation of DPRs in iPSCs, the motor neurons of *C9orf72* ALS patients, and *C9orf72* HRE transgenic mice [113].

Genome-wide CRISPR-Cas9 screens have identified DEAD-box helicase 3 X-linked (DDX3X) as a suppressor of the repeat non-AUG translation. DDX3X directly binds and inhibits sense repeat translation and prevents the formation of DPRs in *C9orf72* ALS patient-derived neurons. Overexpression of DDX3X decreased DPRs, improved NCT, and increased the survival of neurons [114,115]. However, the effectiveness of CRISPR-Cas9-mediated repeat degradation in both experimental animals and clinical subjects should be further investigated.

#### 5.1.3. Small Molecules

Modulators of DPRs have been identified by high-throughput screening. The HSP90 inhibitor geldanamycin and the aldosterone antagonist spironolactone were reported to downregulate DPRs via proteasomal degradation and autophagic clearing pathways, respectively [116]. In addition, the protein kinase K (PKA) inhibitor H89 decreased endogenous DPRs in motor neurons derived from *C9orf72* ALS patients. The in vivo efficacy for ALS should be further investigated.

Interestingly, the beta-blocker propranolol has been demonstrated to protect neurons against p-GA toxicity. In primary neurons overexpressing toxic p-GA, propranolol efficiently reduced aggregate accumulation and increased neuronal survival. The mechanism was associated with enhanced lysosomal degradation of aggregates [117].

Type I protein arginine methyltransferases (PRMT) inhibitors can protect neurons against arginine-containing DRP-induced toxicity in NSC-34 neurons [118]. Similarly, in *C9orf72* knockout homozygous and hemizygous iPSC-derived motor neurons, p-GR dose-dependently induces neurotoxicity. The application of MS023, a PRMT inhibitor, significantly increases neuronal survival and partially rescues neurons from p-GR insult [119].

Tranilast is a drug for asthma and has been demonstrated to activate nonsense-mediated mRNA decay (NMD), which cleaves mRNA substrates to inhibit the translation of defective or harmful proteins [120]. Arginine-rich dipeptides were shown to inhibit NMD, leading to an accumulation of the substrate of NMD in *C9orf72* HRE Drosophila and a decrease in processing body formation, which inhibits NMD. The application of tranilast reduces the abundance of processing bodies and thereby activates NMD and attenuates p-GR-induced neurotoxicity, suggesting that activating NMD holds the potential for treating ALS.

In addition, upstream frameshift 1 (UPF1) is involved in RNA decay. Overexpression of UPF1 reduced neurotoxicity induced by p-GR and p-PR [44] and decreased p-GP abundance but did not affect NMD [121]. The results suggest that targeting UPF1 is also a potential therapeutic strategy for ALS.

### 5.2. Targeting Oxidative Stress

Targeting oxidative stress has also been proven to be effective. Either genetically downregulating the oxidative stress levels or using chemical inhibitors of oxidative stress attenuates *C9orf72* locomotor deficits [102].

KEAP1/NRF2 signaling regulates the oxidative stress pathway. Kelch-like ECH-associated protein 1 (KEAP1) senses the stress and negatively mediates nuclear factor erythroid 2-related factor 2 (NRF2), which activates antioxidant and detoxification genes. Inhibition of the KEAP1/NRF2 pathway by dimethyl fumarate significantly reduces *C9orf72*-related oxidative stress, decreases ROS levels, and reduces motor deficits [102].

### 5.3. Autophagic Modulators

The attempted effectiveness may also be obtained by improving autophagy using autophagic activators. Autophagy inducers, such as rapamycin or apilimod, prevent motor degeneration [122].

Apilimod dimesylate is an inhibitor of phosphatidylinositol-3-phosphate 5-kinase (PIKfyve) that plays a role in endosomal trafficking and autophagy [123]. Apilimod dimesylate increases the number of endosomes and lysosomes in *C9orf72* mice. In a phase 2a clinical trial of treatment with apilimod dimesylate in patients with *C9orf72* HRE, the levels of p-GP in CSF were significantly decreased, and no drug-related serious adverse response was found over the 24-week period of the trial [124].

### 5.4. Targeting Neuromuscular Junction (NMJ)

Both p-PR and p-GA disrupt the NMJ transmission [102,125]. Muscle-specific receptor tyrosine kinase (MuSK) regulates the cell signaling at the NMJ, transferring cell signals between motor neurons and skeletal muscles. P-PR has been demonstrated to interact with Agrin, which interacts with LDL receptor-related protein 4 (LRP4) in the postsynaptic muscle membrane [126]. Agrin is a proteoglycan that plays a role in developing NMJ during embryogenesis and in the organization of postsynaptic differentiation [127]. The disruption of the interaction between Agrin and LRP4 downregulates the activation of MuSK and impairs NMJ transmission.

### 5.5. Targeting NCT

Manipulating the expression of nuclear pore proteins to restore normal nucleocytoplasmic activity can improve outcomes of ALS. Recently, nuclear import receptor β-karyopherin (Kapβ2) was found to interact with p-GR, significantly decreasing cell death in p-GR-expressing neurons [128]. P-PR also interacts with Kapβ and disrupts the NCT [129], suggesting that raising the expression of nuclear import receptors, like Kapβ2, is a potential new avenue for antagonizing the development of neurodegeneration in *C9orf72* ALS. In contrast, inhibition of exportin 1 by KPT-276O prevents nuclear export and significantly attenuates neurodegeneration in the *C9orf72* fly model [35].

### 5.6. Targeting Inflammation

The effects of NLRP3 inhibitors have been investigated in DPR-induced cell toxicity. The non-steroidal anti-inflammatory drugs flufenamic acid and dimethyl fumarate, which are used for multiple sclerosis, can prevent the p-GR-induced activation of the NLRP3 inflammasome in macrophages and reduce the release of IL-1β [63]. In addition, the JAK inhibitor ruxolitinib has been shown to prevent inflammatory response and elevate STAT1 in C9ORF72-deficient cells and mice [130].

To conclude, the efficacy of treatment by targeting different pathogenesis is not yet satisfactory; in one aspect, a single target intervention may not be effective enough to prevent the development of ALS because of the multiplied nature of the underlying mechanisms. In addition, the pathological mechanisms that are associated with *C9orf72* variants only cover a fraction of ALS patients’ causes; solely targeting *C9orf72* variants and their downstream targets could not benefit all ALS patients. Systematic consideration of all the possible causes of ALS and finding the primary or initial mechanism might facilitate finding more effective therapeutic targets for ALS. Otherwise, the therapy may be individualized with specific targets that are dependent on the initial pathogenic causes of the ALS.

## 6. Conclusions and Future Directions

*C9orf72* HRE induces toxic gain by generating RNA foci and DPRs and results in functional C9ORF72 deficiency, which initiates pathogenesis to mediate the progression of ALS. Various pathological mechanisms underlying C9ORF72 HRE have been identified.d The thorough elucidation of C9ORF72 HRE-induced pathogenesis implies that *C9orf72* HRE is a potential therapeutic target for ALS. Although targeting *C9orf72* HRE and associated downstream pathological pathways has been investigated as a possible therapy for ALS, the outcomes are far from satisfactory. Therapy directly targeting HRE has not obtained any clinical benefit, suggesting the complicated pathogenesis of ALS. More studies will be essential to elucidate the pathogenic mechanisms underlying HRE in familial and sporadic cases. Based on insightful elucidation of the ALS pathological process, more effective therapeutic targets and drugs for ALS are anticipated to be developed.

Most research on *C9orf72* HRE exploring pathogenic mechanisms focuses on one aspect. However, the pathological process due to toxic gain of function, loss of function, and their downstream cascades might have crosstalk. Finding common pathways in the pathological process will pave the way to finding more effective targets in ALS. In addition, familial and sporadic ALS have similar characteristic features in gene variations and pathology. Although this review mainly described the mechanisms associated with *C9orf72* HRE, oxidative stress and neuroinflammation are two interacting factors that play essential roles in ALS, which have been described here and elsewhere. Which event occurs first, and which pathways are secondary events? What is the most common initiating mechanism that leads to gene mutations and non-genetic pathogenic outcomes? Targeting the most upstream process that is involved in developing ALS should be the future direction for the therapy of ALS.
